# Foodborne Botulism: Clinical Diagnosis and Medical Treatment

**DOI:** 10.3390/toxins12080509

**Published:** 2020-08-07

**Authors:** Davide Lonati, Azzurra Schicchi, Marta Crevani, Eleonora Buscaglia, Giulia Scaravaggi, Francesca Maida, Marco Cirronis, Valeria Margherita Petrolini, Carlo Alessandro Locatelli

**Affiliations:** Poison Control Centre and National Toxicology Information Centre—Toxicology Unit Istituti Clinici Scientifici Maugeri IRCCS, Maugeri Hospital, Pavia Via Salvatore Maugeri, 10, 27100 Pavia, Italy; azzurra.schicchi@icsmaugeri.it (A.S.); marta.crevani@icsmaugeri.it (M.C.); eleonora.buscaglia@icsmaugeri.it (E.B.); giulia.scaravaggi@icsmaugeri.it (G.S.); francesca.maida@icsmaugeri.it (F.M.); marco.cirronis@icsmaugeri.it (M.C.); valeria.petrolini@icsmaugeri.it (V.M.P.); carlo.locatelli@icsmaugeri.it (C.A.L.)

**Keywords:** botulism, diagnosis, treatment, food, toxicity, Poison Center, poisoning, intoxication, rehabilitation

## Abstract

Botulinum neurotoxins (BoNTs) produced by *Clostridia* species are the most potent identified natural toxins. Classically, the toxic neurological syndrome is characterized by an (afebrile) acute symmetric descending flaccid paralysis. The most know typical clinical syndrome of botulism refers to the foodborne form. All different forms are characterized by the same symptoms, caused by toxin-induced neuromuscular paralysis. The diagnosis of botulism is essentially clinical, as well as the decision to apply the specific antidotal treatment. The role of the laboratory is mandatory to confirm the clinical suspicion in relation to regulatory agencies, to identify the BoNTs involved and the source of intoxication. The laboratory diagnosis of foodborne botulism is based on the detection of BoNTs in clinical specimens/food samples and the isolation of BoNT from stools. Foodborne botulism intoxication is often underdiagnosed; the initial symptoms can be confused with more common clinical conditions (i.e., stroke, myasthenia gravis, Guillain–Barré syndrome—Miller–Fisher variant, Eaton–Lambert syndrome, tick paralysis and shellfish or tetrodotoxin poisoning). The treatment includes procedures for decontamination, antidote administration and, when required, support of respiratory function; few differences are related to the different way of exposure.

## 1. Introduction

Botulinum neurotoxins (BoNTs) are the most powerful natural toxins and are mainly related to *Clostridia* species. These BoNTs can produce a life threatening neuroparalytic syndrome: the “botulism”. Classically, the clinical syndrome of human botulism is characterized by an acute, afebrile, symmetric descending flaccid paralysis. From the clinical point of view, this severe intoxication can be an emergency for which is required a prompt diagnosis and an early identification of sources. Moreover, every case of botulism may be also a public health emergency in case of suspected commercial product ingestion and immediately upon suspecting the diagnosis, the clinician should report the suspected case to Ministry of Health or to the national reference Agencies.

There may be different routes of exposure that characterize different forms of botulism: foodborne, infant and adult intestinal, wound, iatrogenic and inhalation botulism [1,2]. Botulism of unknown source is also mentioned by some authors [3]. All this different forms are clinically characterized by the same syndrome, due to the toxin-induced neuromuscular paralysis. Foodborne botulism is the most frequent form in EU and is the result of the ingestion of preformed BoNT-complexes in food [1]. In all different forms the toxic mechanism is related to effects of a specific xenobiotic, the BoNTs. There are seven known neurotoxins (types A–G), among them, the types A, B and E (rarely F) are toxic for humans, while type C and D mainly cause disease in animals, however human botulism cases have been described in literature as reviewed elsewhere [4]. Type G toxin was suspected in a case of wound botulism [5]. Recently, a chimeric BoNT type FA or HA (also called BoNT/H) was identified in a bivalent *C. botulinum* Bh strain responsible for infant botulism as well as type X was identified in a *C. botulinum* capable of producing type B toxin and isolated from an infant botulism case. In addition, BoNT-like toxins (BoNT/Wo and BoNT/En (BoNT/J) have been described [6].

Botulism patients are considered noncontagious and do not require isolation, but exposure derived from hypothetical aerial dispersion (e.g., terrorist attack) could be a potential source of contamination for rescuers.

In any cases, standard precautions should be exercised when evaluating and treating patients. Botulinum toxin cannot be absorbed through intact skin. Toxin can be absorbed through mucosal surfaces, eyes and non-intact skin. No case of person-to-person transmission of botulinum has ever been described, including in patient care settings. Nevertheless, persons exposed to bodily fluids or stool from patients with botulism should be advised of the early signs of botulism and should report for evaluation if these are noted [1].

A first description of nosocomial transmission of *C. butyricum* type E responsible for two cases of Infant Botulism (IB) has been recently described in two patients came from different geographical area. This experience underlines the importance of apply correct procedures to prevent nosocomial transmission of *Clostridium difficile colitis* and to reduce spreading of neurotoxins producing clostridia spores [7].

From a clinical point of view, despite botulism is an “old” and well described disease, it is important to underline that it remains a rare intoxication characterized by difficulties in clinical suspicion. Medical professional should be aware of this intoxication during the differential diagnosis process. On the other hand, an early clinical diagnosis is crucial to manage appropriately the intoxicated patient with supportive and antidote treatment.

## 2. History

The first outbreaks of botulism were described in 1735. After this, another one occurred in Wildbad in the state of Baden-Württemberg, Germany in 1793, when six persons over 13 died, the source of intoxication has been identified in the common consumption of a popular blood sausage (black pudding). After this first well documented outbreak, the cases of sausage intoxication rapidly increased, until Kerner (a German physician) described 230 cases and, together with this, the characteristic clinical syndrome [8]. In parallel, a syndrome with analogous clinical characteristics, called “fish poisoning” was described by some Russian physicians [9].

The name “botulism” was derived in 1870, by the German physician Muller from the Latin word *botulus*, which means “sausage” [10].

In the 19th century, the microbiologist Emilie Pierre Van Ermengem, in response to an extraordinary outbreak in Ellezelles (Belgium) in December 1895, conducted a scrupulous investigation which led to the isolation of the clostridial organism causing the botulism. In particular, after a funeral meal in which was mainly consumed raw-salted ham, twenty-three of 34 participating musicians over the next two days manifested a neuromuscular paralysis (mydriasis, diplopia, dysphagia, dysarthria and progressive muscle paralysis): three of them died.

Consequently, Van Ermengem defined botulism an intoxication and not an infection and established that a spore-forming obligate anaerobic bacterium, “*Bacillus botulinus*”, was responsible for the toxin production [11]. To express better both its spindle shape (*kloster* is Greek for spindle) and its anaerobic metabolism the pathogen name was later changed in *Clostridium botulinum* [12].

## 3. Toxic Mechanism of Human Foodborne Botulism

The foodborne botulism form is well known in humans and is characterized by a neurological toxidrome consequence of the voluntary motor and autonomic cholinergic junctions’ blockade induced by the toxin. In this form, the preformed BoNT is ingested with food.

The different types of BoNT and the quantity ingested do not influence the toxic mechanism that result quite similar, this latest influences instead the onset (time) of the first clinical manifestations and the severity of the toxidrome.

*C. botulinum* and other BoNT-producing clostridia grow and produce toxin only when the food presents conditions that include an anaerobic milieu, a pH > 4.6, low salt and sugar content and a temperature of 4–45 °C [13]. Home-canned foods or traditional local food represent the major source of intoxication. History is extremely important to formulate or confirm the diagnosis of foodborne botulism and can be provided by obtaining (if possible) the last seven-day food history from the patient.

The bont-producing clostridia generate a polypeptide toxin (150,000 Daltons) that acts specifically on neuromuscular junctions and cholinergic sites within the autonomic nervous system (all ganglionic synapses and post-ganglionic parasympathetic synapses) by binding to receptors located on the presynaptic membrane. After that, by endocytosis and through a complex processes [e.g., translocation of light chain (Lc) into the cytosol and cleavage of soluble N-ethylmaleimide-sensitive factor attachment protein receptors (SNAREs), by the metalloproteinase activity of the Lc] the toxin blocks the normal calcium-associated quantal release of acetylcholine from the presynaptic nerve terminals: this process is irreversible [14].

Acetylcholine molecules are normally contained in vesicles at presynaptic level in units named “quanta”. At first stage, acetylcholine vesicles are synthetized in the neuronal soma and subsequently transported at the terminals where they can be recycled several times. At the terminal level, quanta are contained in three different stores: primary store (immediately available) contains around 1.000 quanta and are nearest the presynaptic membrane; at secondary store (or mobilization store) the quanta (10.000) are available in 1–3 s; the tertiary store (reserve) contains more than 100.000 quanta and are located on the axon or neuronal soma. This physiological asset is completely altered by botulinum toxin.

From the clinical point of view, cranial nerves and muscles are primarily involved during the first neurological stage of intoxication. Moreover, the severity of ophthalmoparesis is considered a good indicator of the overall severity and progression of the intoxication. Probably the initial involvement of cranial nerves is due to three main reasons:

(1) the length of the neurons that innervate the cranial muscles is short, with consequent poor tertiary deposit (acetylcholine reserves are located in the axon or in the soma);
(2) facial muscles are continuously active (even during sleep), with a prevalence of phasic fibers, high neurotransmitter turnover and rapid internalization of toxin;
(3) few receptors are present at postsynaptic facial muscular membrane level that, more quickly, suffer from the lack of acetylcholine.

Recovery may occur only by formation of new axon terminals, with the regenerating axon (sprouting) forming contacts at the original synaptic sites. The time of recovery depends on entity of neuromuscular block associated with neurogenic atrophy (chemo-denervation) and on the regeneration speed of nervous terminals and of presynaptic membranes.

Central nervous system is not affected, and the toxin do not cross the placenta [15].

## 4. Clinical syndrome of Human Foodborne Botulism

The commonest form of botulism has historically been the foodborne, consequently the most known and described typical toxidrome of botulism refers to this form.

Despite this, unfamiliarity of clinicians with this rare poisoning may complicate the initial clinical suspicion. In case of clusters involving more than one case with coherent signs and symptoms, the clinical suspicion for botulism is easier to make [16,17].

The onset time of the botulism toxidrome may vary depending on patient, type and quantity of toxin ingested resulting in the occurrence of symptoms within 12 to 36–48 h after ingestion of contaminated food [18] delayed in some cases up to 10–15 days after ingestion. These differences in the time of presentation can make diagnosis difficult.

Concerning the different type of BoNT, it is well known that BoNT/A is the one that gives the most severe toxidrome and these patients will most likely require intubation [16]; type-E is instead related with a shortest incubation period, while patients with type-B botulism show the longest.

Considering all these characteristics, the aim of the specific treatment is to prevent the gastrointestinal absorption and the bind between the BoNTs and the neuromuscular junctions. Others additional treatment can be added to prevent or limit the in situ production of toxins in forms other than foodborne botulism (e.g., wound and intestinal forms) [15].

Although gastrointestinal disturbances are common, about 30% of patients may not present any abdominal/gastrointestinal signs or symptoms [19]. When present, the early gastrointestinal signs and symptoms consist of nausea, vomiting, abdominal discomfort, pain and diarrhea and are frequently linked to the presence and ingestion of other bacteria or their toxins in the improperly preserved food. These symptoms often precede or accompany the neurological toxidrome and, once these first gastrointestinal symptoms are resolved about 70% of patients manifest constipation.

Autonomic symptoms, such as dry mouth or throat, may be the earliest clinical signs of intoxication and are frequently erroneously interpreted as pharyngitis. Postural hypotension has been reported as autonomic disturbance.

Classically, the overt form of foodborne botulism is characterized by an acute (afebrile), symmetric descending flaccid paralysis. Sensory system is not involved as well as intellectual functions. Clinical manifestations always begin in the bulbar musculature, densely innervated by the IX, X, XI and XII cranial nerves.

As neurological syndrome, the absence of cranial nerves involvement at first stage of poisoning exclude botulism. The main symptoms are dysarthria (frequent), rhinolalia, dysphonia and dysphagia (as involvement of IX nerve) preceded by gastrointestinal discomfort (such as diarrhea followed by constipation) and frequently associated with autonomic dysfunction (i.e., dry or sore mouth and throat) in patients with normal mental status and reflexes, without sensitivity disorders [15]. These complaints are often underestimated. The most characteristic clinical examination of a patient with a typical toxidrome consequently shows diplopia, blurred vision, mydriasis (often fixed), lateral rectus palsy, external ophthalmoplegia and bilateral ptosis that may be prominent.

In severe cases, the clinical features may worsen rapidly to respiratory failure (e.g., without gasping or agitation due to muscle paralysis) and, despite administration of supportive and specific therapy, mechanical ventilation is required.

The sixth cranial nerve palsy may be the initial neurological manifestation of type B botulism, and patients with evidence of a third cranial nerve palsy eventually developed respiratory insufficiency [20].

Oculography studies showed multiple hypometric saccades that induced authors to postulate that the toxin limits the duration of saccadic burst innervation to the extraocular muscles [21]. Pupillary reactions may be present at first clinical evaluation and even months after motor recovery may persist abnormal [22]. Nystagmus is only occasionally noted.

In summary, a neurological syndrome mainly due to a cholinergic blockade may be present at the first evaluation; visual disturbances, dysarthria, dysphagia and dry or sore mouth and throat are the four most specific neurological symptoms [18,19]. If the patient has a mild form of botulism, clinical manifestations stops after the first symptoms with a gradual clinical resolution of the same.

In the most severe cases instead, life-threatening complications may occur because of the prolonged extensive flaccid paralysis: respiratory dysfunction caused by either upper airway obstruction (the weakened glottis tending to close during attempted inspiration) or diaphragmatic weakness. In fact, the most frequent complication in the first botulism phase is airways obstruction or aspiration pneumonia.

The time in which mechanical ventilation is necessary ranges from one to eight weeks with cases in which patients are ventilator-dependent for 7–8 months. The mean period of ventilator support varies according to the BoNT type and is 58 days for the type-A and 26 days for the type-B. As consequence of the prolonged ventilator use, complications such as nosocomial infection can be observed [18].

After this, most patients have residual symptoms at one-year follow-up, including easy fatigability, exertional dyspnea [23], loss of responsiveness to postural change (orthostatic hypotension), hypothermia, alterations in the resting heart rate and urinary retention [24]. The recovery process may take months to be completed and stems from new motor axon twigs that sprout and reinnervate paralyzed muscle tissue.

A recent systematic review of 405 cases of foodborne and wound botulism reported from 1932 to 2015 showed that 86% were foodborne botulism, in 65% of cases associated with an outbreak. At hospital admission, an involvement of at least one cranial nerve was reported for 93% of patients. Only 7% of patients presented with absence of cranial nerve palsies and only with gastrointestinal symptoms, weakness and respiratory distress to the first emergency department (ED) medical evaluation: the cranial nerve involvement appeared in the following 1–3 days in 2/3 of these patients [25]. Cranial nerve alterations were associated with dysphagia (65%), weakness (50%), diplopia (49%) vomiting (44%) and ptosis (37%). Including all foodborne and wound botulism cases, 46% of patients required mechanical ventilation during hospitalization. The median reported duration (data available for 70 patients) was 26.5 days (range, 1–298 days). The reported mortality rate is 25%, but most death occurred before 1980 (case fatality ratio was 40%–60% during 1932–1979 vs 9% during 1980–2015): the decline in mortality rate (for all BoNT types) is primarily due to development of increasingly advanced techniques in supportive and respiratory intensive care and perhaps to a greater knowledge of the problem with consequent early recognition and prompt administration of antitoxin. The incubation period was found shorter in the group with fatal outcome [1 day (range, 0.2–8 days) vs 1.5 days (range, 0.1–12 days)]. Excluding the cases of type F foodborne botulism, that the highest percentage of patients with respiratory distress and mechanical ventilation support resulted in toxin type A group [25].

## 5. Diagnosis and Differential Diagnosis

The diagnosis of botulism is mainly based on clinical suspicion, as well as the decision to apply the specific antidotal treatment. The role of the laboratory is crucial to confirm the clinical diagnosis, particularly in relation to regulatory agencies and to identify the different BoNTs involved and the source of intoxication.

The rarity of the foodborne botulism occurrence is the basis of the fact that it is often underdiagnosed or misdiagnosed; another reason is that the initial symptoms are not pathognomonic and can be confused with more common clinical conditions, such as stroke, myasthenia gravis, the autoimmune acute demyelinating polyneuropathy known as Guillain–Barré syndrome (Miller–Fisher variant), Eaton–Lambert syndrome, tick paralysis and shellfish or tetrodotoxin poisoning [26]. When the gastrointestinal syndrome is prevalent the suspicion of botulism is more difficult and some cases remain undoubtedly entirely unrecognized [19], mainly in the cases in which the neurological syndrome remain blurred. The diagnosis is easier when physicians are facing a large outbreak. However, is not easy to recognize an outbreak and lots of times the first cases are commonly misdiagnosed. It happens, for example, that an outbreak was recognized, only after that the same food-vehicle was associated with a second cluster of cases [27]. Unfortunately, cases of botulism intoxication frequently occur singularly and is not uncommon, that patients are first referred to different specialists (i.e., oculist, otolaryngologist or neurologist) and only after these evaluations and because the worsening of the clinical picture, to the emergency department.

In some cases, the diagnosis has been made only after death, because the existence of a cluster of cases finally addressed the public health authorities to the diagnosis of a botulism outbreak. Therefore, it is clear how a correct diagnosis of first case of botulism is crucial to ensure the detecting and the best clinical management of subsequent cases and particularly to avoid delay in treatment.

Botulism must be considered as a differential diagnosis whenever a patient present with any kind of weakness (Table 1): Generalized, ocular or oropharyngeal weakness, sometimes associated with a history of acute onset of gastrointestinal dysfunction. Bilateral cranial nerve abnormalities and progression of descending paralyses should raise the suspicion of botulism together with the epidemiological criteria.

For what concerns confirmed diagnosis of an initial botulism suspicion reported to the CDC approximately 10.5% of patients are diagnosed as Guillain–Barré syndrome and 6.2%, and 3% as food poisoning and carbon monoxide poisoning, respectively [19].

The confirmation of clinical suspicion cannot be based on routine laboratory tests that would be normal in absence of other complications. In order to distinguish botulism from other similar diseases some tests can be used: (i) normal cerebrospinal fluid (CSF) may differentiate botulism from Guillain–Barré syndrome (even if a slightly elevated CSF protein level is rarely observed in botulism and—in contrast—the protein level may be initially normal in Guillain–Barré syndrome [18]; (ii) tensilon test permits to differentiate the intoxication from myasthenia gravis and (iii) neuroradiologic studies may rule out stroke or ischemic events [27].

A test named “ice pack test”, consisting in the application of ice to closed eyelids, was first, proposed for the differential diagnosis of ptosis and diplopia in 1979 [28,29]. The test was considered particularly valuable for the diagnosis of ocular myasthenia gravis: it has been reported that its specificity can reach 100%, with a sensitivity of 89% in the evaluation of myasthenic ptosis [30]. However, the medical literature does not report evidence on the response to this test in diseases characterized by presynaptic failure of neuromuscular transmission in which ptosis can occur. A single case report of a positive result in a case of Miller–Fisher syndrome is reported [31]. Positive ice pack test could be due to acetylcholinesterase inhibition induced by cooling [32], but it is also possible to hypothesize a facilitation of presynaptic mechanisms of neuromuscular transmission [33,34]. In fact, a case of botulism intoxication with positive ice pack test and suggesting caution in making diagnosis of botulism using this procedure is reported [35].

As the diagnosis of botulism is essentially clinical, key points to differentiate the Miller–Fisher syndrome (variant of Guillain–Barré syndrome) are reported in Table 2**.**

### Laboratory Investigations

Rapid and reliable detection methods in biologic samples are necessary to support clinicians in rapid diagnosis and to help the surveillance systems in identifying the source of contamination and performing epidemiological analysis of the cluster as potential public health emergency [36].

The laboratory confirmation of foodborne botulism is possible with the detection of BoNTs in clinical specimens or food samples and on the isolation of BoNT producing clostridia from stools. The most direct way to confirm the diagnosis is to demonstrate the BoNTs in the patient’s serum or stool by injecting serum or stool into mice and looking for signs of botulism. Other *Clostridia* may produce cases of botulism, for these reasons, the isolation of other BoNT producing clostridia (e.g., *C. butyricum* and *C. baratii*) must be considered in the criteria for laboratory diagnosis [37].

In vivo mouse lethality bioassay (intraperitoneal injections of mice unprotected and protected with polyvalent antitoxin) is routinely used and considered as “gold standard” to confirm the presence of BoNTs in clinical specimen, food and/or environmental samples eventually linked to botulism outbreak. Type-specific botulinum antitoxin is used to identify the type (typing) of the toxins produced from different strains. Mouse bioassay suffers for several disadvantages (costs, time and animal facilities, dedicated personnel, long turnaround time of 1–4 days).

In vitro methods for detection of BoNTs and neurotoxigenic clostridia have been proposed and validated [38,39,40]. To date, immunoassays for detecting BoNTs, assays for detecting catalytic activity of BoNTs, cell-based assays for detecting biologic activity of BoNTs and nucleic acid based methods have been developed, and some of these methods will replace the in vivo mouse lethality bioassay in the future.

An early sample collection (stool and/or gastric content) before antitoxin administration may increase the likelihood of obtaining case confirmation. In the cases of foodborne botulism reported in USA (1975 to 1988), the toxin was isolated in 37% of sera (126/240 cases), 23% of stool (65/288) and 5% of gastric aspirate (3/63). Time influence the results, for example, if the specimens are collected within 1–2 days the percentage of positive increase (60% of sera, 50% of stool) [16].

A recent epidemiological Italian study (1986–2015) identified 285 laboratory-confirmed incidents involving a total of 421 cases. Serum was tested for 65.3% of patients (275/421) of confirmed food-borne cases and resulted positive only for 20.4% of them (56/275). The remaining cases were confirmed by direct detection of toxins in fecal samples (52 patients) or foods (159 patients). A further 154 foodborne cases presenting with the characteristic clinical picture of botulism were laboratory-confirmed through isolation of BoNT-producing *Clostridia* in fecal samples [1].

Usually, different days (up to four days) are needed to obtain laboratory confirmation of botulism. It is well known that the effect of botulinum antitoxin increased if administered early and it is important to underline that the treatment must be applied before the lab confirmation. In this way a rapid, reliable and sensitive assay for detecting BoNTs will provide really benefits to the poisoned patients, especially when special patients are involved, such as children.

In conclusion, endopep-mass spectrometry assays, electrochemiluminescence (ECL) immunoassays, immuno-PCR and enhanced chemiluminescence-based ELISA each demonstrate high levels of sensitivity, having limits of detection comparable to the in vivo mouse lethality bioassay and can rapidly detect active BoNTs in sera or in other clinical matrices [36,41,42,43].

## 6. Treatment

Key points in the treatment of botulism are (i) the decontamination, (ii) the administration of the specific antidote and (iii) the support of respiratory function if necessary. There are only few differences related to the way of exposure.

### 6.1. Gastrointestinal Decontamination

Once excluded eventually contraindications, gastrointestinal decontamination should be performed in all case of foodborne botulism, in order to remove the spores and toxin from the gut. Most patients manifest the first clinical signs some days after consumption of contaminated meal; for this reason, gastric lavage (or induced emesis) should be considered only in cases in which the ingestion of possible contaminated food is recent. For all other cases, the gastrointestinal decontamination, must be applied if the constipation due to the anticholinergic effect may cause a permanence of the contaminated food in the gastrointestinal tract. In these cases, even if the efficacy in suspected and confirmed cases of botulism intoxication is not clearly demonstrated, upper and lower decontamination with oro-gastric tube and cathartics/whole bowel irrigation could be performed. In order to obtain an effective catharsis is better the use of sorbitol because magnesium salts may exacerbate neuromuscular blockade. The whole bowel irrigation may be challenging because of the ileus toxin-induced; in some cases, neostigmine may be useful in reversing ileus, inhibiting the enzymatic degradation of acetylcholine [44]. Activated charcoal administration is also a treatment option in intoxicated patients because it absorbs BoNT/A in vitro [45].

### 6.2. Antidotes

The main goal of the antitoxin treatment is to neutralize the free circulating toxins still unbound at presynaptic level of nerve endings. Additional point is that antitoxin reduces the involvement of new nerve endings: usually the clinical symptoms may progress for up to 12 h after antitoxin administration before an effect is observed [46]. The type-specific antitoxins are not able to counteract any other antigen.

As a general approach, antitoxin should be administered as soon as suspect of botulism intoxication is made. The antitoxin is effective also in all the other forms of botulism. For example, animal studies involving inhalation botulism demonstrated that early administration, after an aerosolized release of botulinum toxin (lethal concentration), may be effective [47].

Equine-derived antitoxin (since 1970), is the unique antidote available. To date, antidote efficacy is well known in experimental studies in animals. In humans, no randomized controlled studies have been performed to evaluate the action of antitoxin therapy (not ethical approach). To date, the efficacy of antidote is based only by case reports and retrospective studies and clinical experiences.

Morbidity and mortality studies are difficult to perform because of the rarity of the intoxication and of the late diagnosis. The diagnosis is normally performed when the BoNT is already permanently entered and fixed at presynaptic level: in this step antidote is incapable to reverse the endocellular mechanism of the toxin.

Some clinical experience confirmed that the early administration of antitoxin (within 24 h), is more effective in preventing the progression of neurological syndrome and in shortening the duration of mechanical ventilation and intensive care stay [48,49].

Tacket and co-authors analyzed 132 cases of BoNT/A foodborne botulism (1973–1980), considering the effect of the antitoxin therapy on the outcome of patients. Lower fatality rate (10% vs 15%) was registered in patients that received antitoxin within 24 h after the onset of symptoms. In the group of patients that did not receive antitoxin the fatality rate was very high (46%). Patients that received early antitoxin (<24 h) had a median hospital stay of 10 days compared with 41 days for those who received antitoxin >24 h and 56 days for those not treated [48].

Nonetheless, patients may need respiratory support for long period, usually 2–6 weeks even if in some cases may be longer: 58 days and 26 days for botulism due to BoNT/A and BoNT/B, respectively [18]. A prolonged rehabilitation program is also needed for some patients with severe intoxication.

Recently, a study performed by Pavia Poison Center—National Toxicology Information Center (Pavia PC) analyzed 98 confirmed cases [53 males (54%)] of foodborne botulism collected during a seven years’ period evaluating the implicated food, clinical presentation at hospital admission, latency between symptoms—hospital admission—treatment, clinical course, response to the antitoxin administration and the results of the specific laboratory analysis. The average age was 50.14 years (SD 17.9), with a range from 5 to 89 years. The indication for specific antidotal treatment was given in all cases presenting typical clinical symptoms with progression during hospitalization or at the time of evaluation by the Pavia PC clinical toxicologists. The trivalent equine antitoxin treatment was administered to 59 patients (60.2%), on average 63 h (SD 68,5) after the onset of neurological complaints. Patients treated within 24 h of neurological symptoms needed less mechanical ventilation (7/26, 26%) compared to patients treated later (14/26, 53.8%). Five adverse reactions were observed (8.4%): 3 mild ones (rush, fever, vomiting, transient bronchospasm) and 2 serious (severe hypotension). The diagnosis of foodborne botulism was confirmed by laboratory analysis in 65/98 cases (66.4%). Toxin serotype B was more often identified (83.6%), whereas serotype A was identified in 6 cases (12.2%). Patients affected by serotype A required mechanical ventilation in higher percentage (83%) than patients affected by serotype B (19.5%) (*p* = 0.004). One case (untreated with antitoxin) required intensive care for 300 days. Only one fatal case was registered (treated with antidote after 24 h) [50].

Despite the two clinical experience are not comparable, mainly for the different study period, the primary aims, data collected, and type of antidote used, key clinical points related to antitoxin administration are summarized in Table 3.

At current time, limited and historical data are obtainable on the link between dose and the amount of circulating antitoxin in treated patients, antitoxin half-life and the toxin-neutralizing capacity. Half-life of circulating antitoxin was documented at 6.5, 7.6 and 5.3 days for antitoxin type-A, B and E, respectively. Antitoxin quantified at peak serum concentrations are 10–1000 times higher than the concentrations estimated to be necessary to achieve toxin neutralization [51]. Published and unpublished clinical data supported the administration of 1 vial of antitoxin that produce a high level of toxin type-specific antibodies (100-fold greater than that needed to counteract the largest amount of circulating BoNT ever measured) [51]. These data refer to a trivalent equine antitoxin (7500 IU type-A, 5500 IU type-B, 8500 IU type-E of antitoxin in each vial) [46].

In Europe, one of the formulations available is the trivalent equine Fermo-serum^®^ containing a different amount of total antitoxin (Table 4). The quantity of antitoxin per mL is similar in all the EU and previous USA formulations: an important difference is related to the total amount of antitoxin potentially administered as indicated by the manufacturer. In fact, the EU antitoxin is recommended by the producer at a dose approximately 40-fold greater than the heptavalent formulation. Acute or delayed hypersensitivity reactions have been reported and more frequently in patients treated with high doses (more than 4 USA vials of antitoxin), suggesting a dose-related phenomenon [52].

On March 22, 2013, the US Food and Drug Administration (FDA) approved Heptavalent Botulism Antitoxin (HBAT) the first product to treat all serotypes of botulism and previously studied by US Army Medical Research Institute of Infectious Diseases (USAMRIID). HBAT is derived from "despeciated" equine IgG antibodies, which have had the Fc portion cleaved off, leaving the F(ab’)_2_ portions. To date, this is the formulation most present in the world. The recommended dose for an adult is one vial (20–50 mL) of HBAT, administered to the patient as an intravenous infusion (diluted with 0.9% sodium chloride in a 1:10 ratio before use). Skin sensitivity testing are optional. Dosage adjustment is proposed in case of involvement of pediatric patient (weight-based correction). HBAT is formulated to meet a minimum potency level for each antitoxin type expressed as unit based on mouse neutralization assay: A (4500 U), B (3300 U), C (3000 U), D (600 U), E (5100 U), F (3000 U) and G (600 U). The half-life of one vial of HBAT ranges from 7.51 h to 34.20 h depending on the antitoxin serotype. The actual potency of HBAT against the many subtypes and mosaics is not known [53].

The safety and improved clinical outcomes was evaluated in patients treated with BAT during the Investigational New Drug (“compassionate use” IND) study period (2010–2013) [54]. A total of 249 persons aged 10 days–88 years (median, 46 years) were treated with BAT. Of these, 17 (7%) were children (median, 6 years; range, 10 days–17 years). None of the 249 treated patients were pregnant or breastfeeding. Botulism was laboratory or epidemiologically confirmed for 104 (42%) patients. Among the 104 patients, all (n = 33) those treated within 24 h of symptom onset (early treatment) survived, while 90% (64/71) of the treated later survived (not statistically significant). In contrast, early BAT treatment was associated with statistically significant shorter hospital (median, 15 vs. 25 days; *p* < 0.01) and ICU stays (10 vs. 17 days; *p* = 0.04) compared with later BAT treatment. Among the 249 patients, 9% of patients experienced at least one adverse effect BAT-related: fever (3%), rash (2%), chills (1%) and agitation, edema, slight hypertension, nausea (1%). Bronchospasm, chest pressure, diaphoresis, erythema, increased respiratory rate, “jitteriness”, leukocytosis, mild hypotension, tachycardia, urinary retention and vomiting were each reported once among adults treated group. In pediatric group (n = 17), single cases of fever, agitation/anxiety and an experience of “hurting all over”, were described. Only one severe adverse reaction occurred in a 10-year-old boy (29 kg body weight) who manifested severe hemodynamic instability characterized by bradycardia leading asystole started 90 min after the BAT infusion and rapidly resolved after epinephrine administration. BAT infusion was restarted and after 30 min a second episode of severe bradycardia occurred, at this point the administration was definitively stopped (an estimated 73% of the recommended dose was administered overall).

A single case of serum sickness occurred in a 64-year-old man, which occurred 11 days after BAT administration and physician-reported as mild, self-limited serum sickness characterized by myalgia and arthralgia treated with ibuprofen; the principal investigator also determined it as not serious.

Historical data (1967–1977) reported an overall rate of adverse effects (including hypersensitivity and serum sickness of 9–17% and anaphylaxis of 1.9% [52]. However, in the previous decades, the recommended dose was 2–4-fold higher than those currently administered. The incidence of hypersensitivity related to an administration of a single vial is reduced; the risk of serum sickness may be approximately 1%–4% [17].

Throughout the world different formulations are available and a comparison is reported in Table 4.

To date, the botulism antitoxin heptavalent (HBAT) is available and currently used also in EU.

In March 2018 an electronic survey was performed through the European Association of Poisons Centers and Clinical Toxicologists (EAPCCT) with the aim to collect epidemiological data and information on the clinical management, the diagnostic capability and the antidote availability in cases of botulism intoxication in Poison Centers/Poisoning treating facilities (PCs) located in different countries. The survey included 19 items on (i) epidemiological data (registered by PCs during 2015–2017) as well as questions on (ii) availability/location of specific laboratory, (iii) clinical management, (iv) type of antitoxin availability (including dosage/adverse drug reaction) and (v) its location. Fourteen PCs answered to the survey (Austria, Belgium, Czech Republic, Estonia, France, Germany, Greece, Iceland, Ireland, Italy, Poland, Slovenia, South Africa and Switzerland).

Ireland, Estonia, Slovenia and Poland PCs declared no experience with botulism because managed by Infectious Diseases Services, 10 questionnaires were analyzed. Cases of foodborne botulism, infant and adult intestinal botulism and wound botulism were registered by PCs. Specific Labs for diagnosis were available in seven countries (70%), all located in government services (in two countries operative 24H). All PCs, except two, prescribes antidote before the laboratory confirmation. Trivalent equine antitoxin was the unique formulation available and the dosage varied from one to four bottles: no severe acute adverse reactions have been reported. Antitoxin is stocked in PCs/Hospitals/pharmacies and in six countries in strategic stockpiles. In conclusion, PCs experience on botulism is extremely different: some services manage all cases occurring in the country as reference centers, while others refer to Infectious Diseases Services. During the study period (3 years), all forms of botulism have been observed by PCs (including rare forms such as wound and intestinal botulism). PCR diagnosing testing is not routinely available, and in vivo tests remain the gold standard method, even if, accordingly, turnaround time (TAT) is too long to be useful in the first phase of the clinical management. Trivalent equine antitoxin is available in EU, and the administration is safe. On the contrary, the recommended dose varies significantly among countries. Antidote storage in strategic stockpiles may be useful to manage public health emergencies or unconventional events. These data underline the need of a harmonization of management of botulism between PCs would seem appropriate for the future [55].

### 6.3. Supportive Airway Treatment

The milestone of treatment for the cases of botulism intoxication is prompt and supportive care. Because of the high risk of rapid respiratory failure and because respiratory compromise close monitoring of respiration is needed.

As suggested by Arnon and colleagues, in mild cases and when a suspicion of botulism intoxication is made the patients should be put in the reverse Trendelenburg position at 20–25° with cervical support; classically, this position enhance diaphragmatic function decreasing the pressure of abdominal viscera and to reduce the risk of aspiration [47].

### 6.4. Antibiotic Therapy

The antibiotic therapy is not able to interfere with the toxin mechanism of action. In wound botulism form the antibiotic therapy alone remains insufficient; it is indicated when secondary infections are documented. In all forms, aminoglycoside antibiotics and clindamycin may exacerbate the neuromuscular blockade [56].

### 6.5. Experimental Treatments

During the last two decades, several efforts on designing new drugs (e.g., monoclonal antibodies) have been made, especially for blocking the catalytic activity of BoNTs. Efforts have been invested on designing small molecules, peptidic inhibitors, aptamers as well as on testing some natural substances for their anti-botulinum activity [13]. Studies on specific inhibitors effective in preventing the neuroparalytic action of BoNTs (irrespectively of their serotype and subtype) which could be used in poisoned patients without knowing the particular type of BoNT are underway and appear to be promising for the future [13,14,53,57,58,59]. Current drug research efforts have mainly focused on BoNT/A and mainly addressed on light chain proteolytic activity. Development of pan-BoNT inhibitors acting independently of BoNT immunological properties and targeting a common step of the intoxication process seems encouraging. In fact, experimental studies on different chemicals or molecules that can interfere with the different stages involving BoNTs mechanisms are available. The new drugs may act as (i) inhibitors of toxin binding, (ii) inhibitors of toxin internalization and trafficking, (iii) inhibitors if toxin translocation, (iv) inhibitors of the toxin disulfide bond reduction, (v) inhibitors of SNARE cleavage by L-chain and (vi) reversal of BoNTs paralysis [53].

The binding of BoNTs to receptors located at the presynaptic membrane is the first step of the intoxication. Some antagonists of the ganglioside receptors (e.g., quinic, lectins from Limax Flavus and tricum vulgaris, thearubigin) have been identified. Main limitations of these treatments are the serotype specificity and the short therapeutic window. Other drugs interfere with internalization process (mainly mediated by dynamin-dependent endocytic pathways) and intracellular trafficking (e.g., Dyngo-4a, methylamine hydrochloride, bafilomycin A, nigericin, quinolinol).

Another option is to interact with specific intracellular toxic mechanism (e.g., adamantanes, lomofungin, chicoric acid botulin, benzimidazole acrylonitrile). Inhibitors of SNARE cleavage by L-chain metalloprotease have been studied, but, despite the promising results, none of these molecules concluded the way to be considered an effective drug [53]. The last group of molecules considered are those involved in functional recovery of intoxicated nerve terminals. The 3,4 diaminopyridine (3,4-DAP) and analogs, a potassium channel-blocking agent, determine an increase of the presynaptic action potential duration causing an increase Ca^2+^ influx and an increase acetylcholine release [60]. The 3,4-diaminopyridine does not cross the blood–brain barrier to a substantial extent [15] and its efficacy in not established [61]. The only benefit observed regards the improvement in ocular and limb muscle strength, but there has been no benefit on respiratory paralysis [62].

Guanidine has been administered in the past to enhance acetylcholine release, but its application, evaluated also in placebo-controlled studies, failed to improve the clinical course of the intoxication [63]. Steroids, immunoglobulins, chloroquine, plasmapheresis, have been tried in single cases with debated benefit.

## 7. Botulism and Pregnancy

Clinical and experimental data suggests that the large molecular weight of the toxin (150 kDa) not permits the passive diffusion through the placenta [64,65]; no cases of clinical or serum evidence of botulism in infants born from mothers poisoned were observed. Recently, a review analyzed sixteen cases of botulism during pregnancy (11 in the third trimester) and one case during the postpartum period. Ten cases were associated with confirmed or likely foodborne exposure, two cases were finally diagnosed as wound botulism due to heroin use and in five cases the source remain unknown. Eleven women (65%) suffered rapid respiratory failure that required mechanical ventilation and admission to intensive care units. About the outcome, two women died, and two women remained in a persistent vegetative state. Eleven women received antitoxin and nor maternal or neonatal ADR were registered [66]. In fact, pregnancy is not considered a contraindication to antidote administration. Antitoxin has been administered with positive outcome [67]. No cases of congenital botulism or neonatal deaths were reported, and six infants were born prematurely. Despite the lack in clinical experience, the administration of BoNT-A in pregnant women seems to be relatively safe for both the expectant mother and fetus [68,69]. No reports of use of BoNT type A during human lactation have been described. Because the toxin is not expected to be detectable in the systemic circulation, it would not be available for excretion into breast milk. Therefore, the risk to an infant is probably zero. No definitive data are available in case of foodborne botulism.

## 8. Conclusive Remarks

Botulism is characterized by an acute, afebrile, symmetric descending flaccid paralysis. In some cases, may results as a medical emergency for which early identification of sources are important. For these reasons, every case of botulism is also a public health emergency and require immediate report of the suspected case to the ministry of health or national agencies. Foodborne botulism, the best-known form, is the most common in Europe and results from ingestion of preformed toxin mainly in improperly preserved home canned vegetables, fish or meat. Despite this, unfamiliarity of clinicians may complicate the initial clinical suspicion. Foodborne botulism is not contagious, and patients do not require isolation: standard precautions should be used when evaluating and treating patients. Initial clinical manifestations are nonspecific and may include nausea, vomiting and xerostomia and then neurological manifestations appear. The diagnosis of botulism is essentially clinical even if the role of the laboratory permits to confirm the clinical diagnosis, to identify the different BoNTs involved and eventually the source. Recovery results from new motor axon twigs that sprout and re-innervate paralyzed muscle: this process may require months to complete. The treatment of botulism includes (i) gastrointestinal decontamination (if indicated), (ii) antidote (antitoxin) and (iii) eventually respiratory support. Antitoxin neutralize only the free circulating toxins in the blood still unbound to the nerve endings (at presynaptic level). The treatment may be started as soon as the clinical suspicion is made. To date, in EU is available and currently used, the botulism antitoxin heptavalent (HBAT) that result safe and effective. During the last two decades, several efforts on designing new drugs have been made, especially for blocking the catalytic activity of BoNTs. Studies on specific inhibitors effective in preventing the neuroparalytic action of BoNTs are underway and appear to be promising for the future.

## Figures and Tables

**Table 1:** Typical toxidrome signs and symptoms of foodborne botulism.

Medical History	Negative for Infectious Diseases
first evaluation	normal mental status, afebrile
	xerostomia/sore throat and/or dysphagia
	anxiety
	normal cardiac activity (rarely bradycardia)
	gastrointestinal manifestations
	weakness
physical exam	symmetric oculobulbar signs
	symmetric descending neurological alterations

**Table 2:** Differential diagnosis between botulism and Miller–Fisher syndrome (variant of Guillain–Barré syndrome) and suggested treatment.

Key Points for Differential Diagnosis	Miller–Fisher Syndrome	Botulism
Positive history for infectious illness (e.g., flu-like syndrome)	may be	no
Oculobulbar symptoms	present	present (early stage)
Pupils	normal	mydriasis
Trend of neuroparalysis	descending	descending
Deep tendon reflex	absent	reduced or normal
Muscle coordination	abnormal (ataxia)	normal
Paresthesias	present	absent
Symmetry of neurological manifestations	yes	yes
Dysautonomia	present	present
Electrophysiological tests (no pathognomonic pattern)	normal motor and sensory conduction velocities with absent H reflexes, slowed nerve conduction velocities, reduced sensory nerve action potential amplitudes or normal studies	normal sensory nerve action potentials. Low amplitude, short-duration and abundant motor-unit action potentials (BSAPs). Small evoked muscle action potential (MAP) in response to a single supramaximal nerve stimulus in a clinically affected muscle
Cerebrospinal fluid	elevated protein levels (may be normal in early stage)	normal
Treatment	plasmapheresis or intravenous immunoglobulin	antitoxin

**Table 3:** Summary of key clinical points of two studies.

Main Data	Tacket et al. 1984 [48]			Lonati et al. 2015 [50]		
Study Period	1973–1980			2007–2013		
BoNT Involved	Bont A			BoNT B (63%), BoNT A (9%), BoNT BF (2%), BoNT AB (1%); not Identified in 25% of Cases		
n° of patients	134			98		
	n° of patients treated with antidote		no antidote	n° of patients treated with antidote		no antidote
	115 (87%)		17 (13%)	59 (60%)		39 (40%)
time of antidote administration	<24 h	>24 h	–	<24 h	>24 h	–
orotracheal intubation (OTI)	n.a.	n.a.	n.a.	26%	53.8%	7.6%
duration of OTI (days)	n.a.	n.a.	n.a.	13.6 ± 5.6	21 ± 15.5	n.a.
median hospital stay (days)	10	41	56	n.a.	n.a.	n.a.
fatality rate	10%	15%	46%	0%	1% *	0%

note: * BoNT-B, female 89-year-old, severe comorbidity. n.a.: not available.

**Table 4:** Botulinum antitoxin products.

Antitoxin Product	Available from	Formulation	Total Amount of Antitoxin (IU)		Recommended Dosage
Trivalent (A/B/E) equine	Biomed (Kraków, Poland)	vial 10 mL	type-A	5000	1–5 vials
			type-B	5000	
			type-E	1000	
Trivalent (A/B/E) equine	Behring (Marburg, Germany)	bottle 250 mL	type-A	187,500	2 bottles
			type-B	125,000	
			type-E	12,500	
HBAT (botulism antitoxin heptavalent) (A/B/C/D/E/F/G) equine	Emergent BioSolutions, Winnipeg, Manitoba, Canada, Inc.	vial 20–50 mL	type-A	≥4500	Adult ≥ 17 yr: 1 vial Pediatric (1–17 yr): 20%–100% of adult dose Infant (≤1 yr): 10% of adult dose
			type-B	≥3300	
			type-C	≥3000	
			type-D	≥600	
			type-E	≥5100	
			type-F	≥3000	
			type-G	≥600	
